# Ultrasonic Energy Field-Induced Recrystallization-Driven Grain Refinement and Mechanical Property Enhancement in Ti5321G Alloy Manufactured via Laser Melting Deposition

**DOI:** 10.3390/nano16191220

**Published:** 2026-09-27

**Authors:** Mingxia Diao, Chunhuan Guo, Fengchun Jiang, Wei Chen

**Affiliations:** 1 Key Laboratory of Superlight Materials & Surface Technology, Ministry of Education, College of Materials Science and Chemical Engineering, Harbin Engineering University, Harbin 150001, China; 2 Yantai Research Institute and Graduate School, Harbin Engineering University, Yantai 264006, China; 3 Shandong Provincial Key Laboratory of Additive Manufacturing Technology & Equipment, Yantai 264006, China

**Keywords:** ultrasonic energy field, grain structure refinement, recrystallization, laser melting deposition, mechanical property

## Abstract

The influence of the ultrasonic energy field (UEF) on the microstructure and mechanical properties of Ti5321G alloy fabricated by laser melting deposition (LMD) was systematically investigated. The application of UEF promotes the transformation of coarse columnar grains into fine and uniform equiaxed grains, reducing the average grain size from 400.3 μm to 145.2 μm, a reduction of 63.7%. Concurrently, the proportion of high-angle grain boundaries increases, and the crystallographic texture is significantly weakened, with the maximum MUD value decreasing by approximately 51.8%. EBSD analysis reveals that the recrystallization fraction substantially increases from 5.3% to 28.1% upon UEF application. As a result, the yield strength, ultimate tensile strength, and total elongation are improved by 6.2%, 5.2%, and 33.8%, respectively. These property enhancements are mainly ascribed to grain-boundary strengthening from grain refinement and improved strain coordination ability of the equiaxed grain structure. The findings demonstrate that UEF-assisted LMD is an effective approach for achieving simultaneous enhancement of strength and ductility in titanium alloy components.

## 1. Introduction

Laser melting deposition (LMD) is a layer-by-layer additive manufacturing technique that offers significant advantages in fabricating lightweight and complex titanium alloy components [1,2]. Owing to its high material utilization, design flexibility, and near-net-shape forming capability, LMD has emerged as a promising alternative to conventional manufacturing routes for producing high-performance metallic parts. However, the rapid melting and solidification inherent to the LMD process generate steep thermal gradients and complex thermal cycles. These processing characteristics tend to promote the directional growth of columnar grains along the building direction, preferentially aligned with the <001> crystallographic orientation in β-titanium alloys. As a result, a strong crystallographic texture develops, accompanied by significant microstructural heterogeneity and mechanical anisotropy [3,4]. Such anisotropic behavior is particularly detrimental to the reliability and service life of load-bearing titanium components, limiting their widespread adoption in safety-critical industries such as aerospace and biomedical engineering. The coarse columnar grain structure also facilitates intergranular cracking and reduces the overall damage tolerance of the as-deposited material. Consequently, refining the grain structure and promoting the columnar-to-equiaxed transition have become critical scientific challenges for enhancing the mechanical performance of LMD-processed titanium alloys. Achieving a fine, homogeneous, and weakly textured microstructure is essential for improving both the strength and ductility of the final components. Therefore, exploring effective and industrially viable methods for grain refinement is of paramount importance for the further advancement of LMD technology in titanium alloy fabrication.

Many efforts have been made to obtain equiaxed grains instead of coarse columnar grains [5,6,7]. One common approach is the addition of alloying elements, which can induce constitutional supercooling ahead of the solidification front or promote heterogeneous nucleation, thereby facilitating equiaxed grain formation [8]. However, this compositional modification inevitably alters the original alloy chemistry and may introduce undesirable secondary phases, making it unsuitable for high-precision alloy systems where composition control is critical [9]. Alternatively, external field control techniques, such as pulsed arc and electromagnetic stirring, have been employed to refine the grain structure by modifying the solidification conditions [10,11]. Although these methods can improve the microstructure to some extent, they usually require complex auxiliary equipment and additional processing steps, leading to increased manufacturing costs and extended production cycles. Therefore, a simple, cost-effective, and industrially viable grain refinement technique is urgently needed to overcome the inherent limitations of current approaches and to enable the production of LMD-processed titanium alloys with enhanced mechanical performance.

In recent years, ultrasonic energy field (UEF)-assisted additive manufacturing has attracted considerable attention as a promising technique for microstructural control [12,13,14]. It is well established that UEF can effectively refine microstructures, promote the columnar-to-equiaxed transition, and significantly reduce structural anisotropy in metallic components. Despite these recognized benefits, the underlying mechanisms governing microstructural evolution during UEF-assisted LMD of titanium alloys remain inadequately understood. In particular, the role of ultrasound in influencing recrystallization behavior, which is a key process for grain refinement and texture weakening, has not been systematically clarified. Furthermore, there is a lack of comprehensive studies that establish quantitative relationships among grain equiaxed transformation, crystallographic texture evolution, and the corresponding improvements in mechanical properties. This knowledge gap hinders the rational design of UEF parameters for tailored microstructures and optimized performance. Therefore, a systematic investigation into the recrystallization behavior and its interplay with texture and mechanical properties is essential to fully harness the potential of UEF-assisted LMD for high-performance titanium alloy components.

Although previous study (Ref. [4]) has demonstrated that UEF-assisted LMD can effectively refine the grain structure of Ti5321G alloy, the intrinsic relationship among UEF-induced recrystallization, crystallographic evolution, and mechanical performance remains unclear. In our previous work, the grain refinement mechanisms were mainly identified by separating the contributions of solidified-layer recrystallization and melt-pool-related acoustic effects using different UEF configurations. However, the evolution of recrystallization behavior and its contribution to mechanical property enhancement were not investigated.

Therefore, the present study focuses on a different scientific question: how UEF promotes recrystallization during the thermal-mechanical history of LMD and how the enhanced recrystallization behavior regulates microstructure and mechanical properties. Through EBSD-based recrystallization analysis, quantitative grain structure characterization, texture evaluation, and tensile testing, this work establishes the correlation between recrystallized fraction, grain refinement, texture weakening, and strength–ductility enhancement. These findings provide new insights into the role of UEF-induced recrystallization in tailoring the performance of additively manufactured titanium alloys.

## 2. Experimental Procedure

This study employed gas-atomized Ti5321G powder (particle size 53–150 µm). The chemical compositions are presented in Table 1. After being dried in a vacuum oven (120 °C, 2 h), the powder was deposited onto the titanium substrate by LMD. Under constant pressure, the ultrasonic probe sustained uninterrupted contact with the deposited layer and moved in synchronization with the LMD equipment, as depicted in Figure 1. Deposition parameters included a scanning speed of 400 mm/min, laser power of 2700 W, powder delivery rate of 10 g/min, and argon shielding gas at 15 L/min (99.99% purity); ultrasonic frequency of 20 kHz, amplitude of 50% (peak amplitude 20 µm). The ultrasonic transducer was mechanically coupled with the substrate/deposition system during LMD processing. The relative position between the ultrasonic source and melt pool was maintained constant throughout fabrication. The UEF parameters used in this work were selected based on previous studies and preliminary experiments to demonstrate the feasibility of ultrasonic-assisted LMD for Ti5321G alloy [4]. The objective of the present study was not to optimize ultrasonic parameters but to clarify the relationship between UEF application, recrystallization behavior, microstructural evolution, and mechanical properties under a representative processing condition. Unidirectional multilayer deposition was adopted, with the X direction as the deposition direction and the Z direction as the building direction.

Microstructural characterization was performed on the YZ planes of the thin-wall specimens. The morphology and grain boundaries of the β phase were observed using optical microscopy (OM, ZEISS Axioscope 5). The OM samples were sequentially ground, polished, and etched with Kroll solution (5 mL HF, 15 mL HNO_3_, and 80 mL H_2_O).

Electron backscatter diffraction (EBSD) analysis was performed using a Hitachi S3400 scanning electron microscope equipped with HKL Channel 5 software. The measurements were conducted at an accelerating voltage of 20 kV, a step size of 4 µm, an acquisition rate of 10.6 Hz, a working distance of 15 mm, and a sample tilt of 70°. EBSD samples were prepared by mechanical grinding and subsequent ion-beam polishing. The EBSD step size was selected considering the relatively large β-grain size of the deposited Ti5321G alloy. Even after UEF treatment, the average β-grain size remained approximately 145 μm, resulting in more than 30 measurement points across an average grain diameter. Therefore, the selected step size was sufficient for statistical evaluation of grain size, grain-boundary characteristics, texture evolution, and recrystallization behavior. The average EBSD indexing rate was approximately 98.5% for the analyzed regions. Data processing for grain structure and texture analysis was carried out with HKL Channel 5 software. EBSD data were cleaned using standard post-processing procedures.

Uniaxial tensile tests were conducted using an Instron 5565 universal testing machine at a strain rate of 10^−3^ s^−1^. The tensile specimens, with dimensions shown in Figure 1, were extracted from the YZ planes of the thin-wall components along the building direction. For each condition, three specimens were tested, and the average values with standard deviations were reported. Although the number of tensile specimens was limited, the relatively small variations among repeated measurements indicate good testing reproducibility. The gauge surfaces were ground with 3000-grit SiC paper to remove machining marks. A digital extensometer was used to monitor strain during loading. The yield strength was determined using the 0.2% offset method. The specimens investigated in the present work were fabricated independently from those reported in Ref. [4]. Although the same alloy system and ultrasonic-assisted LMD technology were employed, the deposition experiments, specimen preparation, characterization, and mechanical testing were newly conducted for this study.

## 3. Results and Discussion

(1) Microstructures analysis

Figure 2 presents the OM microstructures of Ti5321G alloys fabricated by LMD without and with UEF assistance, respectively. As shown in Figure 2a, the sample fabricated without UEF exhibits a typical coarse columnar grain structure, with grains growing preferentially along the deposition direction (*Z*-axis) and extending continuously across multiple deposited layers. This columnar morphology originates from the high thermal gradient along the deposition direction during the LMD process, which drives epitaxial grain growth from the bottom of the molten pool. Such microstructural features are commonly observed in additively manufactured titanium alloys and significantly affect the anisotropy of mechanical properties. In contrast, Figure 2b shows that the UEF-assisted sample exhibits a fundamentally transformed microstructure. After applying UEF, the coarse columnar grains are significantly refined and transformed into fine, uniform equiaxed grains, which are homogeneously distributed throughout the deposited layers with no obvious directional preference. This indicates that UEF effectively disrupts the directional growth tendency of columnar grains and promotes the columnar-to-equiaxed transition. In summary, UEF-assisted LMD can effectively eliminate columnar grain structures and achieve a uniform and fine equiaxed grain morphology. This microstructural optimization is of great significance for improving the mechanical property anisotropy and achieving synergistic enhancement of strength and ductility in additively manufactured titanium alloys.

Figure 3 presents the EBSD grain morphology maps of Ti5321G alloy processed under conditions without and with UEF, respectively, which are consistent with the OM observations shown in Figure 2. In the absence of UEF (Figure 3a), the microstructure is dominated by coarse columnar grains, which are strongly elongated along the deposition direction (*Z*-axis). In contrast, with the application of UEF (Figure 3b), a fundamental microstructural transformation is observed. The coarse columnar grains are significantly refined and replaced by uniformly distributed fine equiaxed grains, accompanied by a marked increase in the proportion of HAGBs. The rise in HAGB fraction is beneficial for enhancing the grain-boundary strengthening effect and promoting dislocation slip coordination, thereby contributing to improved ductility. Figure 3c,d shows the inverse pole figures (IPFs) of the β phase with respect to the X, Y, and Z axes, which quantitatively reflect the evolution of crystallographic texture using MUD as an indicator of texture formation [15]. Without UEF (Figure 3c), the maximum MUD values for the {001}, {101}, and {111} crystal plane families are 3.52, 3.23, and 3.69, respectively, indicating a pronounced <001> texture, which is a direct manifestation of the preferential growth of columnar grains along the deposition direction. After UEF application (Figure 3d), the corresponding MUD values decrease significantly to 1.89, 1.86, and 1.78, respectively, representing a reduction of 46.3%, 42.4%, and 51.8%, respectively. Moreover, the intensity distribution across the three crystal plane families becomes more uniform, indicating effective texture weakening. This texture modification is attributed to the UEF-induced grain refinement and equiaxed transformation, which eliminate the directional growth advantage of columnar grains and promote a more random crystallographic orientation [16]. In summary, EBSD analysis confirms the microstructure-modulating effect of UEF from three perspectives: grain morphology, grain-boundary characteristics, and crystallographic texture. UEF not only refines the grains but also significantly reduces the texture intensity, thereby establishing a microstructural foundation for mitigating the mechanical property anisotropy of LMD-fabricated titanium alloys. These findings further highlight the effectiveness of UEF as a promising tool for microstructural control in additive manufacturing.

The effect of UEF on grain refinement was quantitatively analyzed by distinguishing low-angle grain boundaries (2–15°, LAGBs) and high-angle grain boundaries (>15°, HAGBs) [17], as presented in Figure 4. In the absence of UEF, the sample exhibits an average grain size of 400.3 ± 30.1 μm, a grain aspect ratio as high as 4.0 ± 1.0, and a columnar grain fraction of 73.5%. These data indicate that the as-deposited microstructure of LMD-processed alloy without ultrasonic assistance is dominated by coarse columnar grains, with a wide grain size distribution and significant anisotropy. After the application of UEF, the average grain size is significantly refined to 145.2 ± 17.2 μm, corresponding to a reduction of 63.7%. The grain aspect ratio decreases to 2.2 ± 0.5. res. Microstructural quantification is based on the classification of Todaro et al. (equiaxed: <2.5 aspect ratio; near-equiaxed: 2.5–3.3; columnar: >3.3) [12]. The proportion of equiaxed grains substantially increases from 8.2% to 52.5%, while the columnar grain fraction sharply drops to 20.2%. The increased proportion of equiaxed grains is beneficial for improving ductility and strain coordination capacity. The above quantitative data further confirm the significant effect of UEF on grain refinement and microstructural homogenization from a statistical perspective. UEF not only substantially reduces the grain size but also promotes the morphological transition from columnar to equiaxed grains and increases the fraction of HAGBs. These synergistic microstructural modifications provide further insights into the relationship between β-grain evolution and mechanical-property enhancement in Ti5321G alloy.

To elucidate the underlying mechanisms by which UEF improves the grain structure of Ti5321G alloy during LMD, the recrystallization behavior was systematically examined under conditions with and without UEF, as presented in Figure 5a,b. Recrystallized grains generally exhibit low internal orientation gradients due to the elimination of deformation-induced lattice distortion, whereas deformed and recovered grains retain higher orientation variations. Therefore, grains were classified according to their internal misorientation values following previously reported criteria for additively manufactured metallic materials [18,19,20]. Based on grains with internal orientation differences, the β phase grains are classified into three types: recrystallized grains (<1.3°, blue), deformed grains (1.3–7.5°, red), and substructured grains undergoing recovery (7.5–15°, yellow) [4]. This classification provides a clear distinction between fully recrystallized, deformed, and partially recovered microstructures. In the absence of UEF (Figure 5a), the recrystallization fraction (RF) is only 5.3%. The recrystallized grains are sparsely and isolatedly distributed. The matrix is mainly composed of substructured grains (yellow). This indicates that during conventional LMD, rapid solidification and complex thermal cycles lead to a high-energy non-equilibrium state. However, the driving force for recrystallization is insufficient. Thus, significant recrystallization hardly occurs. After applying UEF (Figure 5b), the recrystallization fraction significantly increases to 28.1%. Recrystallized grains are uniformly and densely distributed throughout the observed region. This change indicates that UEF effectively activates the recrystallization process. It enables the previously suppressed recrystallization behavior to fully develop. The uniform distribution of recrystallized grains further suggests that the effect of UEF is global rather than localized. This is of great significance for achieving overall microstructural homogenization.

Figure 5c presents a schematic diagram of the mechanism by which UEF promotes recrystallization. It discusses the complete physical process from ultrasonic action to grain refinement. It should be noted that the experimental results directly demonstrate the enhancement of recrystallization behavior and the associated microstructural evolution under UEF. However, the detailed atomic-scale processes involving dislocation motion, rearrangement, and stored-energy variation are proposed based on the established mechanisms of ultrasonic-assisted deformation and require further experimental verification. The recrystallization mechanism associated with UEF can be interpreted from the perspective of stored-energy evolution. During LMD processing, the introduction of ultrasonic energy provides additional mechanical input to the deposited material, which may increase the stored deformation energy and modify the thermodynamic state of the material. The ultrasonic mechanical input may promote defect accumulation and stored-energy evolution during LMD processing. The increased stored energy is considered a possible additional driving force for recrystallization, while stored-energy gradients may facilitate recrystallization nucleation and grain-boundary migration. Consequently, the enhanced recrystallization tendency induced by UEF leads to an increased recrystallized fraction and contributes to the refinement of β grains [6,21,22]. Consequently, UEF enhances the recrystallization tendency, resulting in an increased recrystallized fraction and refined β grains. Nevertheless, the quantitative relationship between ultrasonic energy input, defect evolution, stored-energy variation, and recrystallization kinetics requires further investigation.

As Ti5321G is a near-β titanium alloy, the possible influence of β-to-α phase transformation during cooling should be considered. In this work, no α-phase was identified within the detection capability and spatial resolution of EBSD in the analyzed regions. Therefore, the grain structures characterized in this study are considered to correspond predominantly to the β phase. Therefore, the grain sizes of 400.3 μm and 145.2 μm represent β-phase grain structures rather than α-phase morphology.

It is acknowledged that equiaxed grains and high-angle boundaries alone cannot uniquely demonstrate recrystallization because similar features may also originate from phase transformation or recovery processes. Therefore, recrystallization identification was not based solely on grain morphology or grain-boundary characteristics. The increase in equiaxed grain fraction was considered as supporting evidence, while the recrystallized fraction was quantitatively determined from EBSD orientation-based analysis (Figure 5a,b). Furthermore, the recrystallization process during LMD should be considered as a coupled thermo-mechanical process. UEF introduces additional energy and promotes stored energy accumulation, which provides the necessary conditions for subgrain evolution and boundary migration. Therefore, the observed β-grain refinement is attributed to UEF-assisted recrystallization during the LMD thermal history.

(2) Mechanical properties

Figure 6 provides a comparative analysis of the mechanical properties of Ti5321G alloys fabricated by LMD with and without UEF assistance. The results clearly demonstrate that the introduction of UEF leads to substantial improvements in both strength and ductility. Specifically, the yield strength (YS) increases from 659.5 ± 10.0 MPa to 700.5 ± 8.8 MPa, representing an increase of 6.2%. The ultimate tensile strength (UTS) rises from 865.1 ± 18.0 MPa to 910.7 ± 9.1 MPa, corresponding to a gain of 5.2%. More notably, the total elongation (TE) shows a remarkable enhancement from approximately 8.0% ± 0.8% to 10.7% ± 0.5%, reflecting an improvement of 33.8%. The simultaneous increase in strength and elongation indicates that UEF-assisted LMD can partially overcome the typical strength–ductility compromise observed in the investigated Ti5321G alloy. However, this conclusion is based on the selected UEF condition and the tested building direction; broader validation requires investigation of different processing parameters and loading orientations. It should be noted that only three tensile specimens were tested for each condition due to the limitation of sample preparation. The variations among repeated tests were relatively small. For yield strength, the increase from 659.5 ± 10.0 MPa to 700.5 ± 8.8 MPa is substantially larger than the corresponding standard deviations, indicating a reliable strengthening effect induced by UEF. Similarly, the increase in total elongation from 8.0% ± 0.8% to 10.7% ± 0.5% exceeds the experimental variation, suggesting improved deformation capability. Three independent tensile specimens were tested for each condition. Considering the limited sample size, 95% confidence intervals were calculated to evaluate the uncertainty of the measured properties. The results indicate that the UEF-treated samples exhibit higher strength and elongation values than the untreated samples, although further experiments with larger sample populations are required for comprehensive statistical validation.

To further evaluate the effectiveness of the present UEF-assisted LMD strategy, a comparison between the present work and recently reported ultrasonic-assisted LMD titanium alloys was conducted, as shown in Figure 6c. It should be noted that the mechanical properties reported in different studies are influenced by variations in alloy composition [23,24,25,26], additive manufacturing parameters, specimen orientation, and testing conditions. Therefore, the comparison mainly aims to evaluate the overall strength–ductility balance rather than directly rank individual materials. The present Ti5321G alloy exhibits a competitive combination of yield strength and total elongation among ultrasonic-assisted additive manufactured titanium alloys. 

The strengthening and deformation mechanisms discussed below are interpreted based on the experimentally observed microstructural characteristics, including grain refinement, recrystallization behavior, grain-boundary modification, and texture evolution.

The significant improvement in tensile properties is mainly attributed to the UEF-induced recrystallization during LMD. The high-frequency mechanical vibration introduced by UEF alters the solidification dynamics of the molten pool. It also generates periodic strain energy accumulation in the solidified deposits. This energy input promotes recrystallization. It significantly refines the grain size and greatly increases the grain-boundary density. The newly formed fine grains are characterized by a high fraction of HAGBs. The improvement in strength is considered to be partially associated with grain refinement-induced strengthening. The reduction in β-grain size increases grain-boundary density and may enhance resistance to dislocation motion according to the Hall–Petch relationship. According to the Hall–Petch relationship, the refined β-grain structure is expected to increase resistance to dislocation motion and contribute to the observed yield strength enhancement [27]. However, the measured yield strength improvement may also involve contributions from other microstructural factors, including changes in dislocation structures, residual stress state, crystallographic texture, and defect distribution. Since these factors were not quantitatively separated in the present study, the contribution of grain-boundary strengthening should be considered as one of the important mechanisms rather than the sole dominant factor. In addition, the refined grain structure promotes more uniform dislocation distribution and interaction among grains. This effectively avoids local strain concentration and thus improves the overall plastic deformability.

Meanwhile, UEF-induced recrystallization significantly promotes the columnar-to-equiaxed transition. This is another key microstructural mechanism for improving ductility. Equiaxed grains exhibit better strain coordination ability due to their near-isotropic crystallographic characteristics. During tensile loading, equiaxed grains distribute strain more uniformly. They effectively relieve local stress concentration and delay the onset of necking and fracture. In addition, the formation of equiaxed grains eliminates the strong crystallographic texture caused by columnar grains. This leads to a more random grain orientation distribution. The randomized texture is expected to contribute to a more homogeneous mechanical response. It also makes crack propagation paths more tortuous and complex. This increases the energy consumption for crack growth. The tortuous crack paths require higher fracture energy. This significantly enhances the fracture resistance and overall toughness of the material [4]. It should be noted that tensile properties in this study were evaluated only along the building direction. Therefore, the influence of UEF on mechanical anisotropy is inferred mainly from microstructural indicators, including grain morphology and texture evolution. Although the weakened texture and increased fraction of equiaxed grains suggest a potential improvement in mechanical isotropy, tensile testing along multiple orientations will be necessary to quantitatively evaluate anisotropic mechanical behavior in future work. For near-β titanium alloys, β→α transformation may occur during cooling or subsequent thermal cycles. Although no detectable α phase was observed in the present EBSD analysis, the possible presence of nanoscale α precipitates cannot be completely excluded. Therefore, the influence of phase transformation on mechanical behavior should be considered as a potential factor.

Fractographic observations, presented in Figure 7, provide further validation of the mechanical testing results. The UEF-treated sample exhibits a fracture surface characterized by uniformly distributed fine dimples, indicative of microvoid coalescence and a ductile fracture mode. In contrast, the sample fabricated without UEF displays a relatively flat fracture surface with cleavage facets, which is characteristic of brittle fracture. The transition from brittle to ductile fracture with UEF application highlights the efficacy of ultrasonic treatment in enhancing the intrinsic ductility of the alloy, corroborating the measured improvement in total elongation. The weakened texture may improve deformation compatibility by reducing the preferential activation of specific crystallographic orientations during plastic deformation. This effect, together with grain refinement and increased recrystallized fraction, may contribute to the enhanced ductility observed after UEF treatment. However, the influence of texture randomization on crack propagation behavior and fracture resistance requires further investigation using dedicated fracture characterization methods.

In summary, the UEF-assisted LMD process achieves multiple synergistic effects through recrystallization. These include grain refinement, an increased fraction of high-angle grain boundaries, columnar-to-equiaxed transition, and texture randomization. These microstructural modifications collectively contribute to the improved mechanical response. Grain refinement and an increased HAGB fraction enhance strength through grain-boundary strengthening, while the equiaxed grain structure and weakened texture improve strain accommodation during deformation. It should be noted that the present study mainly focuses on the relationship between UEF application, recrystallization behavior, microstructural evolution, and mechanical properties. Although the observed increase in recrystallization fraction supports the proposed mechanism of UEF-enhanced recrystallization, direct experimental evidence regarding dislocation evolution and stored-energy variation, such as quantitative dislocation density, was not obtained in this work. Therefore, these nanoscale mechanisms are proposed based on established theories of ultrasonic-assisted deformation and require further investigation.

(3) Residual stress considerations

In addition to microstructural refinement and recrystallization behavior, residual stress evolution may also influence the mechanical response of additively manufactured titanium alloys. However, the contribution of residual stress to the observed property enhancement was not quantitatively evaluated in the present study. During LMD processing, the repeated thermal cycles, rapid solidification, and constrained shrinkage may generate considerable residual stresses. Ultrasonic energy input may influence residual stress evolution through high-frequency mechanical oscillations, possible plastic accommodation, and defect redistribution during deposition [28,29]. However, whether UEF-induced residual stress relaxation contributes significantly to the measured mechanical-property improvement remains unclear.

In the present study, although residual stresses were not directly measured, the observed increase in recrystallized fraction, grain refinement, and texture weakening suggests that UEF alters the deformation and thermal history of the deposited material. These microstructural modifications may be associated with changes in residual stress distribution during deposition. Nevertheless, because residual stresses were not directly measured in this work, their contribution to the improved tensile properties cannot be separated from the effects of grain refinement, recrystallization, and texture modification. Therefore, residual-stress relaxation should be considered as an unresolved factor requiring further investigation.

(4) Comparative discussion with previous work

It is essential to clearly distinguish this study from our previous work (Ref. [4]). In Ref. [4], four UEF configurations (N, S, L, and S+L) were designed. These configurations were used to systematically decouple the two grain refinement mechanisms induced by UEF: recrystallization in the solidified layer and cavitation/acoustic streaming in the liquid melt pool. That study identified the active mechanisms. However, it did not investigate the recrystallization process or discuss the physical origin of UEF-induced recrystallization from the perspective of stored-energy evolution.

In contrast, the present study addresses a fundamentally different scientific question. It focuses on the driving force for UEF-enhanced recrystallization and quantitatively reveals its contribution to the mechanical properties. A simplified experimental design, with and without UEF, was adopted. Combined with systematic EBSD-based recrystallization analysis, the study established quantitative correlations between the increase in recrystallized fraction (from 5.3% to 28.1%) and multiple performance improvements. These include a 63.7% reduction in grain size, a 51.8% decrease in texture intensity, and a 33.8% increase in elongation. More importantly, UEF was found to increase the stored energy of the material. This provides an additional thermodynamic driving force for recrystallization, thereby promoting microstructural optimization and improving mechanical properties. The progressive relationship between recrystallization origin, microstructure, and properties was not addressed in Ref. [4].

Therefore, Ref. [4] focused on identifying the two grain refinement mechanisms, whereas the present work elucidates the origin of the recrystallization driving force and its role in property enhancement. The two studies are complementary rather than repetitive. This work provides new insight into how recrystallization enables the simultaneous improvement of strength and ductility in UEF-assisted laser melting deposition of titanium alloys.

(5) Industrial perspectives and limitations

Although the present study demonstrates the effectiveness of UEF-assisted LMD in refining microstructure and improving mechanical properties, several issues should be considered for future industrial implementation. In the current experimental setup, the ultrasonic probe is integrated with the deposition system and moves synchronously with the laser deposition head while maintaining a constant relative distance from the deposition zone. This configuration ensures stable ultrasonic energy input during fabrication and avoids the synchronization issue between a stationary ultrasonic source and a moving deposition process.

However, when extending this technique to large-scale manufacturing of complex components, maintaining uniform ultrasonic energy distribution over a larger deposition area may become challenging. The effective ultrasonic field is influenced by factors such as component geometry, probe arrangement, energy attenuation, and deposition trajectory. Therefore, UEF-assisted LMD shows potential for industrial applications, although further studies are required to address challenges associated with process scale-up.

Moreover, although the current system achieves stable relative motion between the ultrasonic probe and deposition head, industrial implementation may require more advanced process integration strategies, such as adaptive control of the probe position, ultrasonic parameters, and deposition trajectory, especially for components with complex geometries or variable deposition paths.

These challenges do not affect the feasibility demonstrated in the present work but represent important considerations for translating UEF-assisted LMD from laboratory-scale fabrication to industrial-scale additive manufacturing.

## 4. Conclusions

In summary, this study systematically investigated the effect of UEF on the microstructure and mechanical properties of Ti5321G alloy fabricated by LMD. The main conclusions are drawn as follows:

(1) UEF-assisted LMD significantly refines the alloy microstructure. The average grain size decreases by 63.7%, the proportion of equiaxed grains increases from 8.2% to 52.5%, and the crystallographic texture is notably weakened. This microstructural refinement is attributed to UEF-induced recrystallization, with the recrystallization fraction rising from 5.3% to 28.1%.
(2) UEF enhances the recrystallization behavior during LMD, as demonstrated by the increased recrystallized fraction, refined grain structure, and modified grain-boundary characteristics. The enhanced recrystallization may be associated with UEF-induced thermo-mechanical effects and possible stored-energy evolution.
(3) Consequently, the yield strength, ultimate tensile strength, and total elongation are improved by 6.2%, 5.2%, and 33.8%, respectively. These enhancements arise from grain-boundary strengthening, the superior strain coordination of equiaxed grains, and the random grain orientation that prolongs crack propagation paths. The UEF-assisted LMD process therefore provides an effective approach for balancing strength and ductility in Ti5321G alloy components.
(4) It should be emphasized that the scientific objective and contribution of the present study are distinct from those of previous work (Ref. [4]). Ref. [4] focused on identifying the dual mechanisms of UEF-induced grain refinement. In contrast, this study further investigates the nature of UEF-enhanced recrystallization from the perspective of possible stored-energy evolution. The observed increase in recrystallized fraction suggests that UEF modifies the deformation and thermal history of the deposited material, which may contribute to stored-energy accumulation and provide an additional driving force for recrystallization. This promotes microstructural optimization and improves the mechanical properties. These findings provide new insights into a key issue that was not addressed in previous studies.

## Figures and Tables

**Figure 1:**
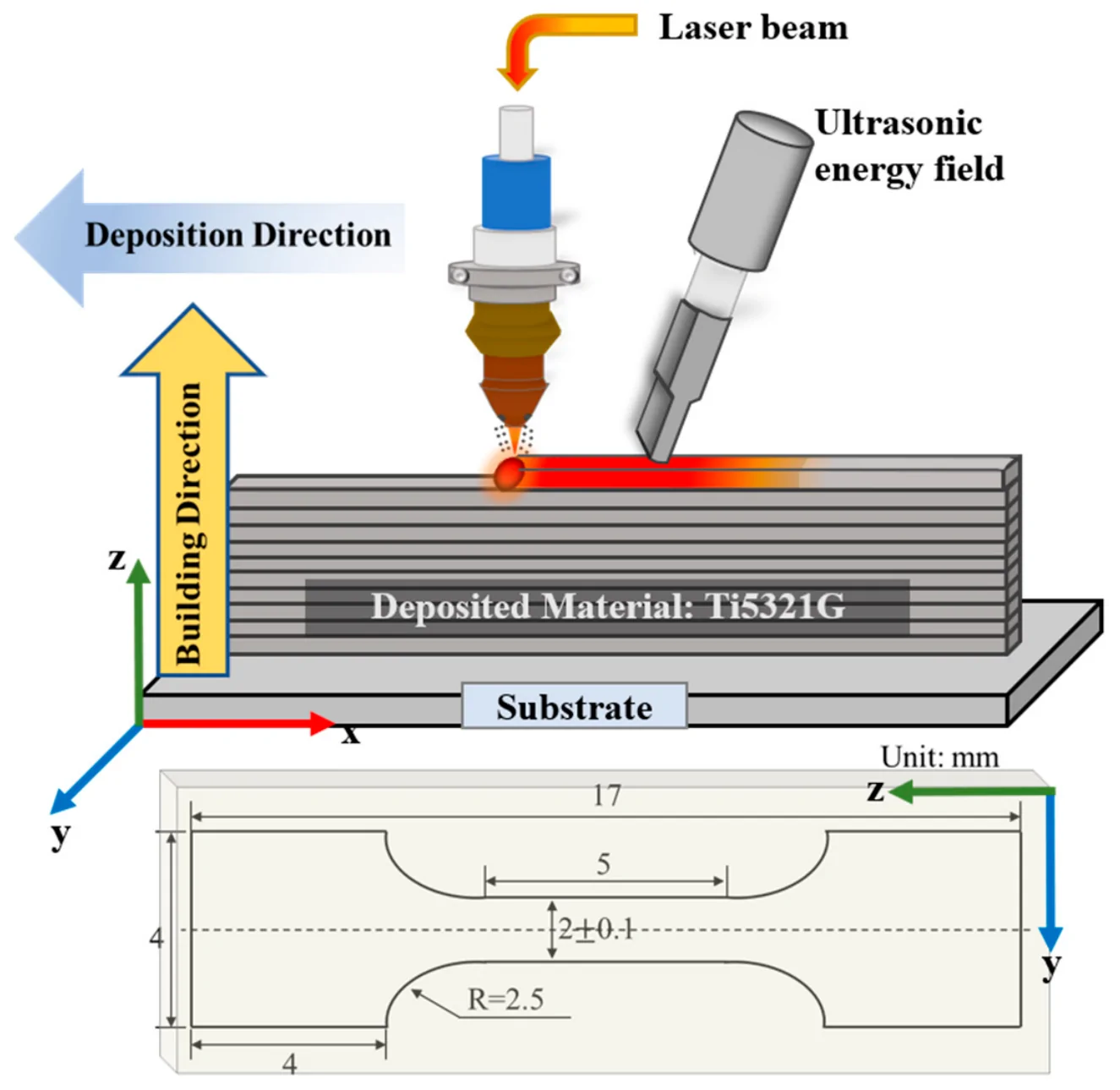
Schematic illustration of LMD with assistance of UEF along with the dimensions and orientation of the plate tensile samples.

**Figure 2:**
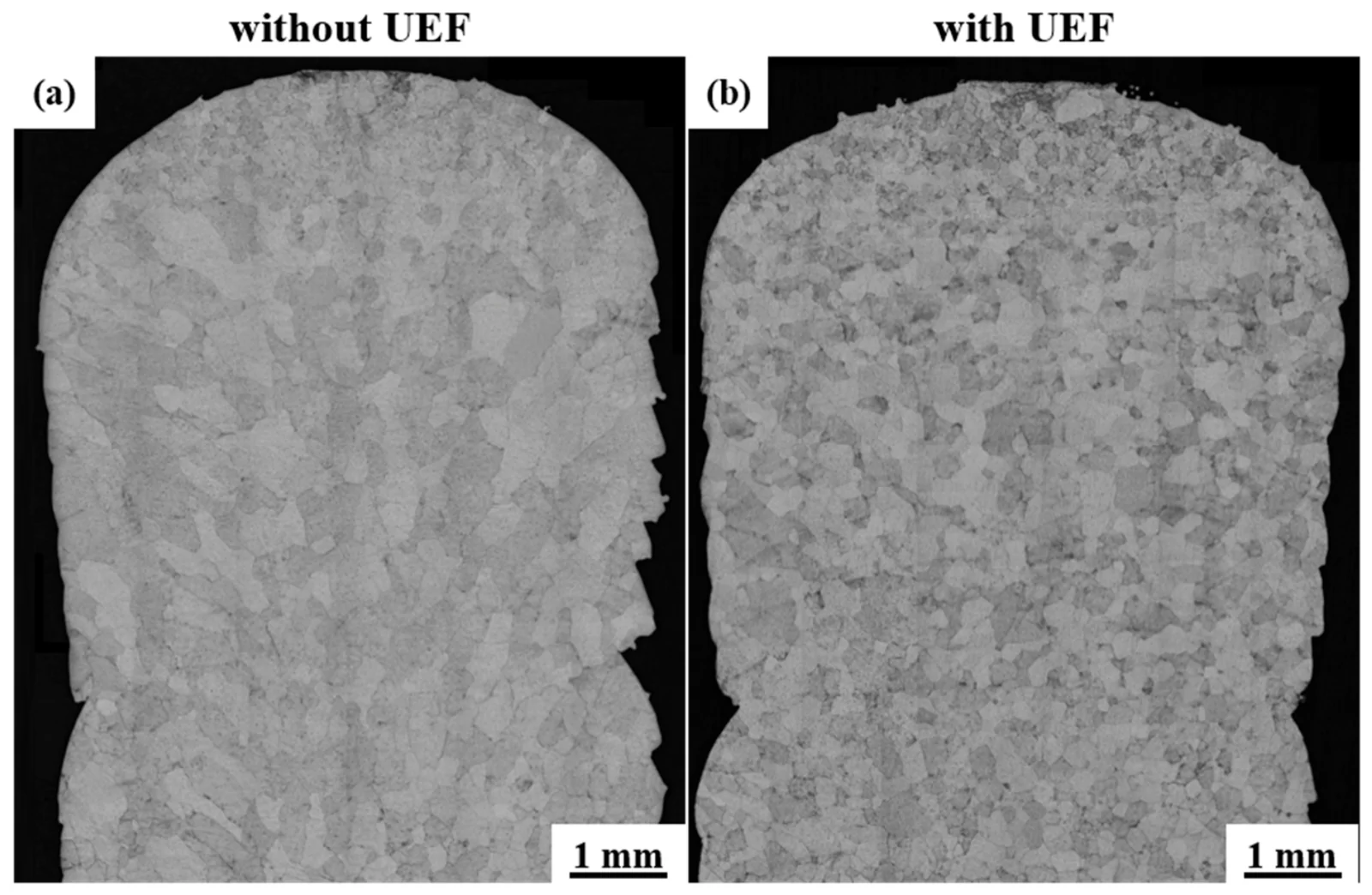
OM images of Ti5321G alloys manufactured via LMD: (**a**) without UEF; (**b**) with UEF.

**Figure 3:**
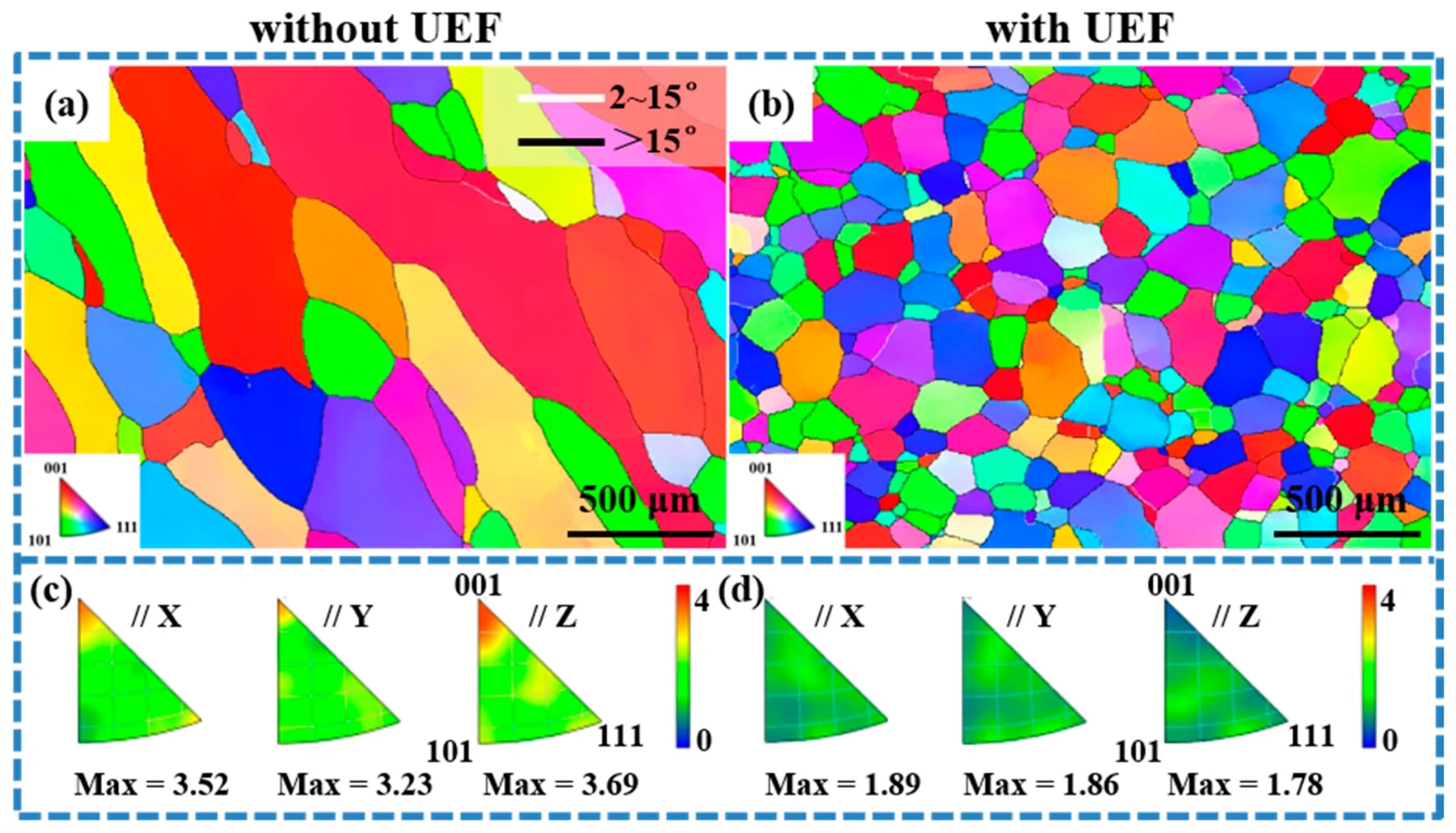
Microstructures of Ti5321G alloys manufactured via LMD: (**a**,**b**) β grain morphologies along the *z*-axis (building direction) without and with UEF, respectively; (**c**,**d**) IPF maps without and with UEF, respectively.

**Figure 4:**
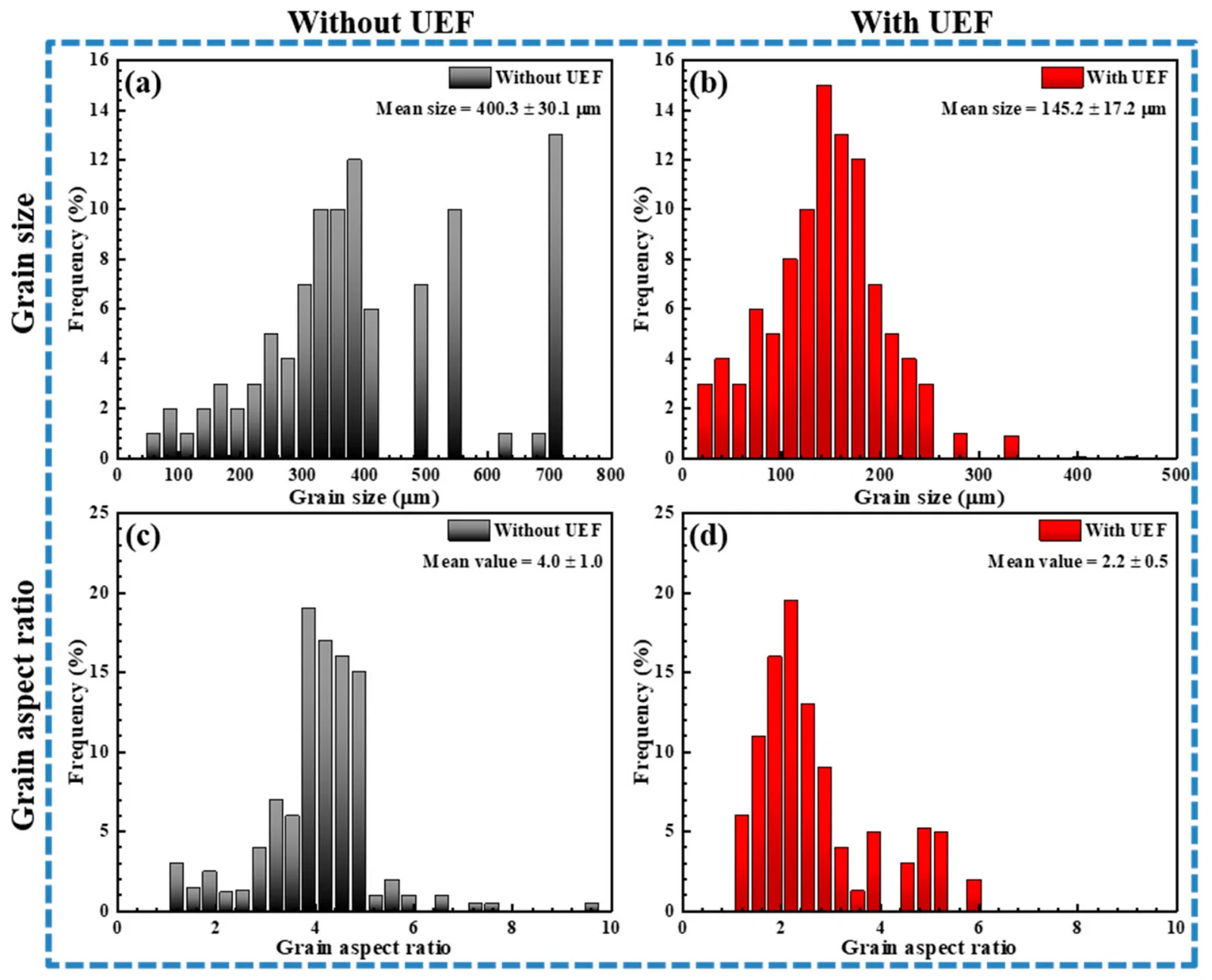
Quantitative determination of the β-phase grain structure in Ti5321G alloys manufactured by LMD: (**a**,**b**) grain size without and with UEF; (**c**,**d**) grain aspect ratio without and with UEF.

**Figure 5:**
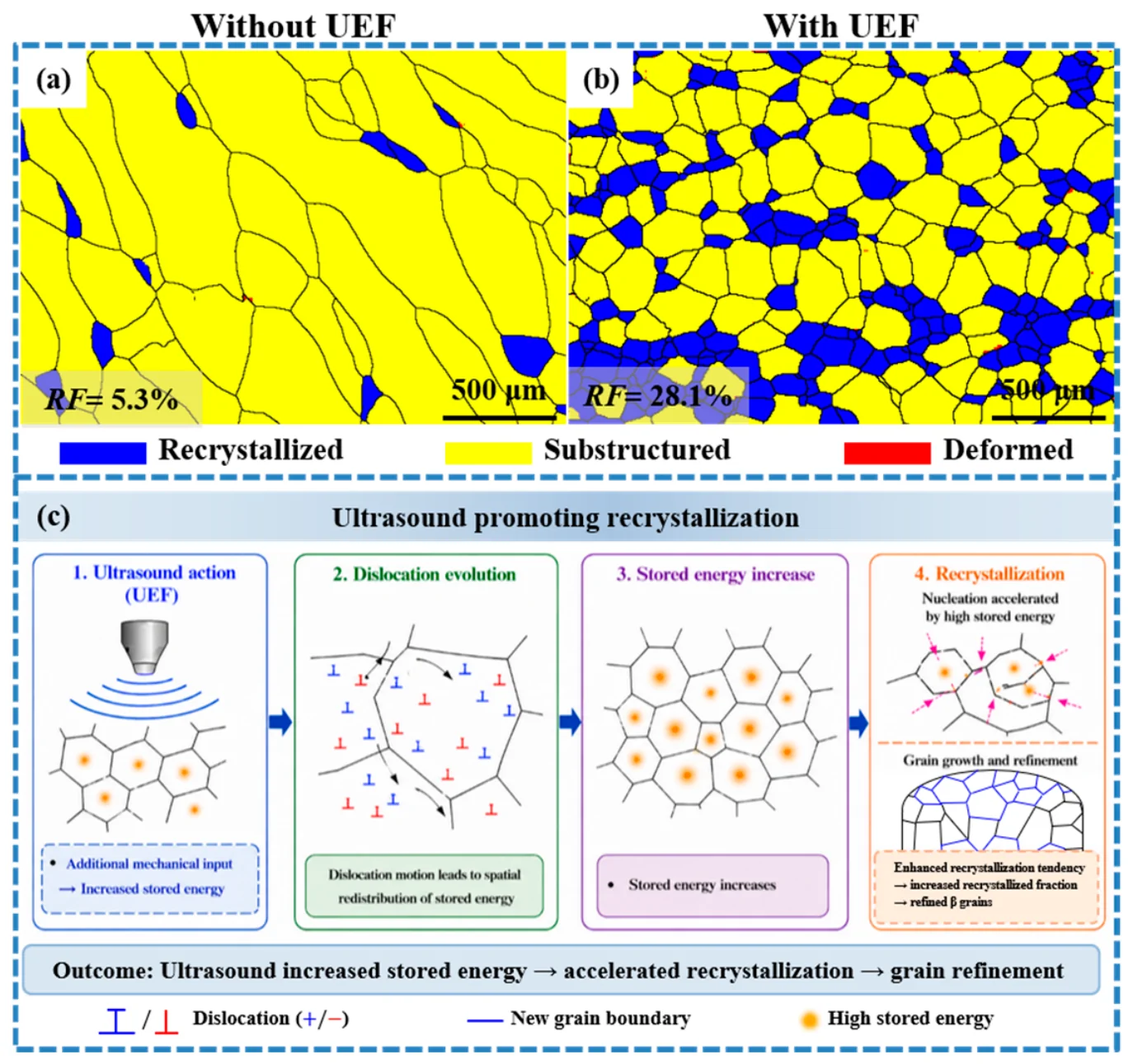
UEF-promoted recrystallization in Ti5321G alloys manufactured via LMD: (**a**,**b**) RF maps without and with UEF; (**c**) Mechanistic diagrams.

**Figure 6:**
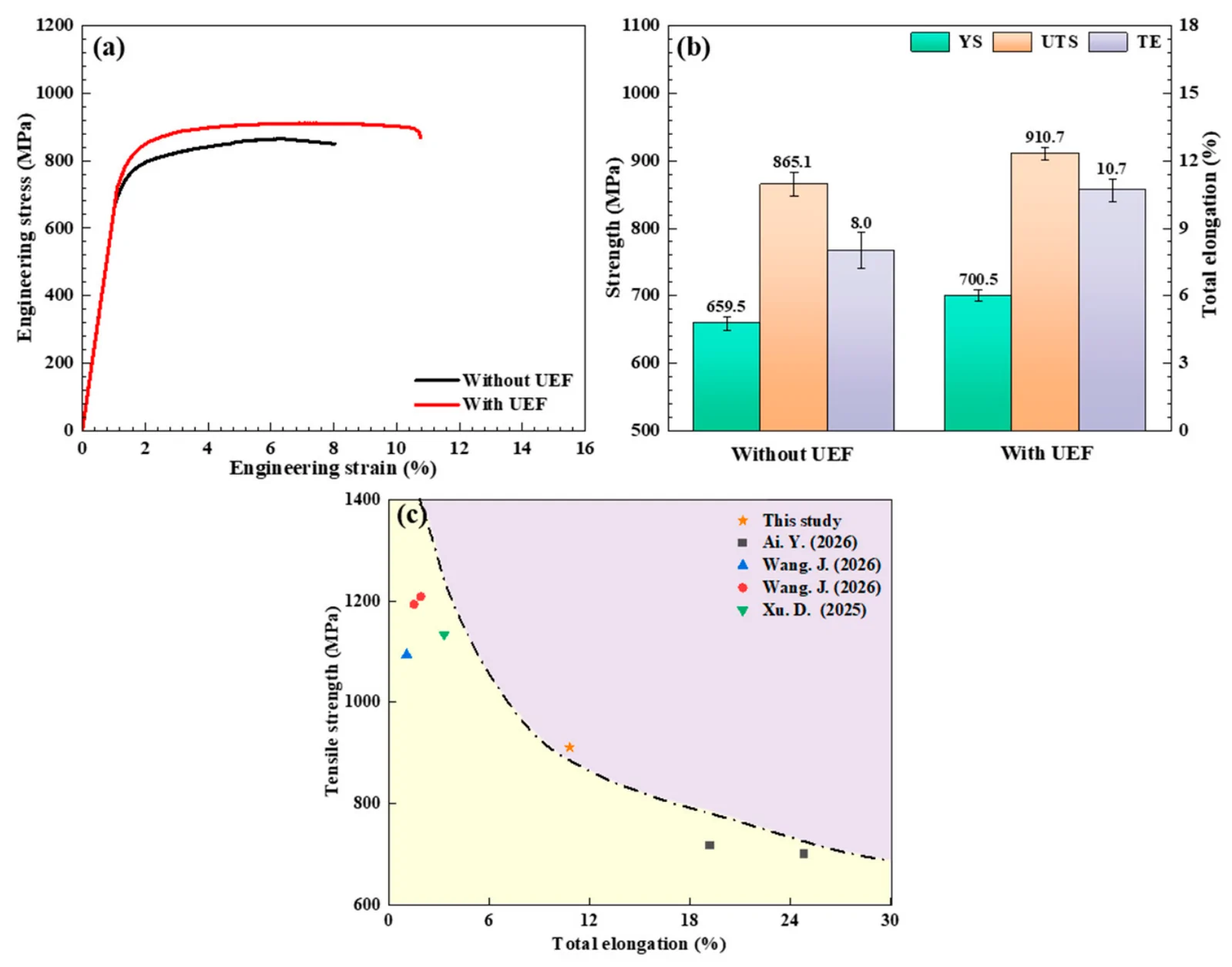
Mechanical properties and SEM fractography of tensile specimens of Ti5321G alloys manufactured via LMD: (**a**) engineering stress–strain curve; (**b**) histograms comparing the YS, UTS, and TE (error bars represent standard deviations obtained from three independent tensile tests); (**c**) Comparison of yield strength and total elongation between the present work and recently reported ultrasonic-assisted LMD titanium alloys.

**Figure 7:**
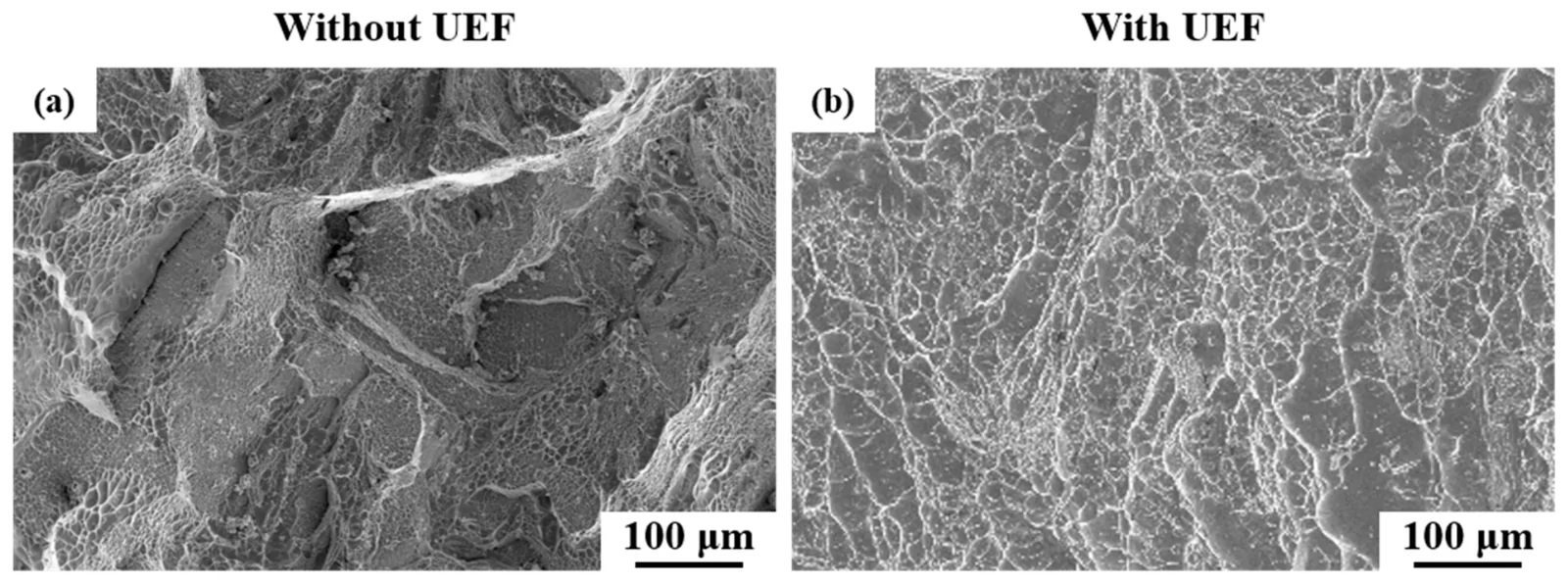
SEM fractography of Ti5321G alloys manufactured via LMD: (**a**) without UEF; (**b**) with UEF.

**Table 1:** Chemical Composition of the Ti5321G Alloy Powder (wt.%).

Al	V	Nb	Mo	Zr	Ti
5.27	2.97	1.05	4.13	1.89	Bal.

## Data Availability

Data will be made available on request.
